# Synergistic effect of sorption and photocatalysis on the degree of dye removal in single and multicomponent systems on ZnO-SnO_2_

**DOI:** 10.1007/s11356-021-18044-7

**Published:** 2021-12-21

**Authors:** Olga Długosz, Anita Staroń, Paulina Brzoza, Marcin Banach

**Affiliations:** Faculty of Chemical Engineering and Technology, Cracow University of Technology, Warszawska St. 24, 31-155 Cracow, Poland

**Keywords:** Nanoparticles, Sorption, Photocatalysis, Dyes, Multicomponent system, ZnO-SnO_2_

## Abstract

The paper presents the photodegradation process of one-, two- and three-component dye mixtures by ZnO-SnO_2_ nanoparticles. After 60 min of running the processes, the dye removal efficiencies of 76.44, 72.69, 62.43, 77.00 and 92.46% for MB, RB, TB, MO and YQ degradation, respectively, were obtained. For binary and ternary systems, dye removal efficiencies for all cases exceeded 70%. When the binary and ternary dye mixtures were tested, the photodegradation efficiencies of ZnO-SnO_2_ were similar to those of the single mixtures, indicating that this material could be used in industrial applications in the future. The focus of the study was to investigate the effect of sorption on photodegradation efficiency and the presence of both cationic and anionic dyes on their degradation efficiency under UV light. The significance of the effect of sorption on the degradation efficiency allowing the interaction of the catalyst with the dyes removed was confirmed. The main factor influencing sorption and consequently photocatalysis was the nature of the dye. It was confirmed that the positively charged ZnO-SnO_2_ surface effectively sorbs the dyes and causes their degradation.

## Introduction

Due to the increase of pollutants that are present in water, the use of standard water treatment methods is insufficient. Therefore, new pollutant removal technologies that are effective, economical and environmentally friendly are being intensively sought. One such method is the removal of contaminants from the aqueous phase by photocatalytic degradation of organic compounds in the presence of ZnO nanostructures (Jiang and Pi 2018). The nanoscale zinc oxide can currently be considered one of the most important semiconducting oxides. This is due to its unique properties, including high chemical stability, high electrochemical coupling coefficient, wide radiation absorption range and high photostability (Siddiqi et al. 2018). Zinc oxide has a wider, compared to TiO_2_, energy band which is about 3.3 eV, high binding energy (60 meV) and high thermal and mechanical stability (Promnopas et al. 2015). Nanoparticles and nanocomposites used for water and wastewater treatment are gaining popularity. Apart from TiO_2_, zinc(II) oxide is one of the most commonly reported semiconductors used in photodegradation processes.  Modification of ZnO can be achieved by addition of SnO_2_, α-Fe_2_O_3_, WO_3_, ZrO_2_ (Davari et al. 2017; Sorbiun et al. 2018; Ebrahimi et al. 2019; Qi et al. 2020). Two important semiconductors, ZnO and SnO_2_, have attracted considerable attention due to their unique conduction properties, mechanical properties, simple microstructure and corresponding conduction and conduction band gap (Karpuraranjith and Thambidurai 2017a; Isabel Bento Rovisco et al. 2020).

Complex structures of photocatalysts are obtained using various methods (Enesca and Andronic 2021). The literature reports, among others, the use of TiO_2_ to form a heterogeneous structure with LaNiO_3_ perovskite by sol–gel method and confirmation of the photocatalytic activity of the obtained material (Zhang and Jaroniec 2018; Chen et al. 2020). The fabrication of two- (CuxS/SnO_2_) and multicomponent (TiO_2_/CuxS-CuO/SnO_2_ and ZnO/CuxS-CuO/SnO_2_) thin film tandem structures by robotic spray pyrolysis representing a potential material for UV–Vis degradation of pollutants was also described (Enesca et al. 2015). The co-precipitation method produced a BiOBr/BiORBr-x heterojunction with excellent photocatalytic activity for degradation of many contaminants under visible light (Li et al. 2020). An s-scheme BiOBr/BiOAc1-xBrx heterojunction was also formed at room temperature in which the lifetime of the carriers with stronger redox capability was extended (Jia et al. 2020).

In contrast to the passive removal of pollutants that takes place in filtration processes, sorption, etc., photocatalysis degrades organic pollutants in water or wastewater by oxidation of free radicals and reduction of hydration electrons (Atchudan et al. 2017; Zhang and Zhang 2020). Photocatalysis using solid-state oxide materials is a widespread technique used to remove a wide variety of organic contaminants (Kashinath et al. 2017b). Recently, research has been conducted on advanced oxidation methods. Such methods include heterogeneous solid-state photocatalysis (Ayodhya and Veerabhadram 2018). It enables the efficient generation of hydroxyl radicals for the complete removal of organic contaminants, mainly of pharmaceutical origin. Recent studies have shown that heterogeneous photocatalysis is the most efficient technique for the degradation of dyes (Kashinath et al. 2017a; Chiu et al. 2019; Sun et al. 2020). This method made it possible to completely decompose organic compounds into harmless substances such as CO_2_ and H_2_O.

Much of the research on the photodegradation of organic compounds is based on the analysis of single-component systems. Mixtures of dyes, active compounds, drugs, etc. may have different effects than individual forms and may increase or decrease toxicity (Arshad et al. 2018; Medhi et al. 2019). In industrial wastewater treatment processes, including photocatalytic processes, there are various parameters that affect the treatment efficiency. These parameters include catalyst mass, dye concentration, pH, light intensity, photocatalyst type and temperature. They influence the rate of decomposition of individual components, the form of their occurrence and the activity of the photocatalyst itself. Additional parameters omitted from the analysis are the presence of additional compounds, the analysis of interactions between individual components and between the components and the catalyst surface (Rovisco et al. 2021).

Among the organic pollutants found in wastewater, dyes are an important group. Dyes are widely used in various branches of industry. They can be found, for example, in the textile, tanning and fur, paper and wood industries, construction and furniture, household and automotive chemical industries. Dyes are contaminants which are difficult to remove during the treatment process. Dyes may be highly toxic to aquatic organisms and may interfere with certain chemical and biological processes such as photosynthesis and oxygen solubility (Karpuraranjith and Thambidurai 2017b; Saeidi et al. 2019). A significant proportion of dyes belong to the group of azo dyes. Due to their complex chemical structure, the molecules are resistant to light, oxidising conditions and decomposition by microorganisms.

The aim of the study is to indicate the possibility of using ZnO nanoparticles modified with tin(IV) oxide as a photocatalyst in the processes of decomposition of dyes in aqueous solution under UV light in single and multicomponent systems. The novelty in the paper includes an attempt to explain the mechanism of interaction between sorption and photodegradation processes and the analysis of dye decomposition from multicomponent solutions, i.e. solutions that are actually found in wastewater. In this way, it is possible to better describe the removal processes of organic pollutants and maximise their removal efficiency by combining the sorption and photocatalytic properties of the material used for their removal. The research carried out in this work is a response to the current problems of pollution of the aqueous environment by dyes. The obtained system was tested for photocatalytic properties in the processes of dye decomposition in aqueous solution under UV light by analysing the interactions between the individual components present in the systems.

## Materials and methods

### Materials

For the preparation of modified zinc oxide (ZnO NPs), the following reagents were used: ZnSO_4_∙7H_2_O (98%, POCH), SnCl_4_ (> 99%, Sigma Aldrich) and Na_2_CO_3_ (> 99%, Sigma Aldrich). The photocatalytic properties of the materials were verified by dye degradation processes (Table 1) The dyes were selected due to the difference in particle size (molar masses ranging from 327.2 to 872.9 g/mol) and the ionic nature of the dyes (anionic, cationic and neutral). The dyes were from Sigma-Aldrich. Solutions were prepared by dissolving the compounds in deionised water.

**Table 1:** Dyes used for photodegradation

Nr	Dye	Abbreviation	Molar mass [g/mol]	Character	$\lambda$[nm]
1	Methylene blue (MB)	MB	319.9	Cationic	664
2	Yellow quinoline (YQ)	YQ	477.4	Neutral	412
3	Rhodamine B (RB)	RB	479.0	Cationic	554
4	Trypan blue (TB)	TB	872.9	Anionic	590
5	Orange methylene (MO)	MO	327.3	Anionic	464

### Methodology

In order to obtain ZnO-SnO_2_ NPs, zinc oxide was obtained as the particle core in the first step. For this purpose, 10 ml of 2 M Na_2_CO_3_ solution was added to 20 ml of 1 M ZnSO_4_ salt solution in the presence of ultrasound. The precipitated metal hydroxides were dehydrated in a microwave reactor at 180 °C to obtain ZnO NPs. The material was filtered, washed with water, and dried at 105 °C. In the second step, 0.768 g of ZnO NPs was weighed, and a solution of SnCl4 was added in a volume of 20 ml 1 M such that SnO_2_ constituted 10%mas in the final product. A 2 M solution of Na_2_CO_3_ was added to the suspension in the presence of ultrasound in a stoichiometric ratio. The precipitated Sn(OH)_4_ hydroxide deposited on ZnO oxide was dehydrated in a microwave reactor at 180 °C to obtain ZnO-SnO_2_ nanoparticles. The material was filtered, washed with water, and dried at 105 °C.

### Photodegradation of dyes in systems

The study of the photodegradation of dyes in the presence of a photocatalyst was carried out by adding 200 mg of catalyst to 60 ml of 18 mg/dm^3^ dye solution. After 60 min of sorption in the absence of light, the samples were exposed to 365 nm ultraviolet radiation (UV light). The lamps were placed 20 cm above the samples. The entire system was protected to prevent exposure to other light sources. The calculated value of the light irradiance was 5.3 mW/cm^2^. During the study of photodegradation processes of binary systems, the concentration of dyes was 9 mg/dm^3^ each, and the concentration of dyes in ternary systems was 6 mg/dm^3^ each, so that the total initial concentration of dyes of the system was constantly 18 mg/dm^3^.

The efficiency of dye removal from solution in single-component systems by photodegradation was determined from the relationship:

1 $$E [\%]=\frac{{C}_{0}-C}{{C}_{0}}\cdot 100\%$$

where $E$ is the decomposition efficiency of the selected dye and ${C}_{0}$ is the initial concentration of the dye. For single-component systems, the amount of dye removed from solution per photocatalyst was also determined:

2 $$R [mg/g]=\frac{\left({\mathrm{C}}_{0}-C\right)\cdot \mathrm{V}}{\mathrm{m}}$$

where $V$ is the volume of the dye solution (ml) and $m$ is the mass of the photocatalyst (g). In multicomponent systems, overlapping of spectra from individual dyes is observed, resulting in a mixture spectrum.

### Methods

The phase composition of the materials obtained was investigated by XRD (Philips X’Pert camera with monochromator PW 1752/00 CuKα). The structure of ZnO-SnO_2_ was investigated by scanning electron microscopy (SEM) with EDS to obtain a mapping of the surface composition of the material. The diffuse reflectance spectroscopy was performed in the range of 200–700 nm. Reflectance spectra were recorded using a UV-2600 spectrophotometer (Shimadzu). The concentration of dyes was determined by spectrophotometry (Rayleigh UV-1800 spectrophotometer). Calibration curves were prepared with the absorbance characteristics of each dye.

The value of zero-point exchange was examined in using dynamic light scattering (DLS) (Malvern Instruments, ZS-90 and Brookhaven, ZetaPALS). To 50 ml H_2_O, 0.1 M NaOH/HCl was added to establish pH in the range of 2 to 12. Such prepared solution (50 ml) was mixed with 25 mg ZnO-SnO_2_ nanoparticles. Suspensions were mixed by 24 h and after the zeta potential in each pH were determined.

## Research results and discussion

### Characteristics of ZnO-SnO_2_ material

**Fig. 1:**
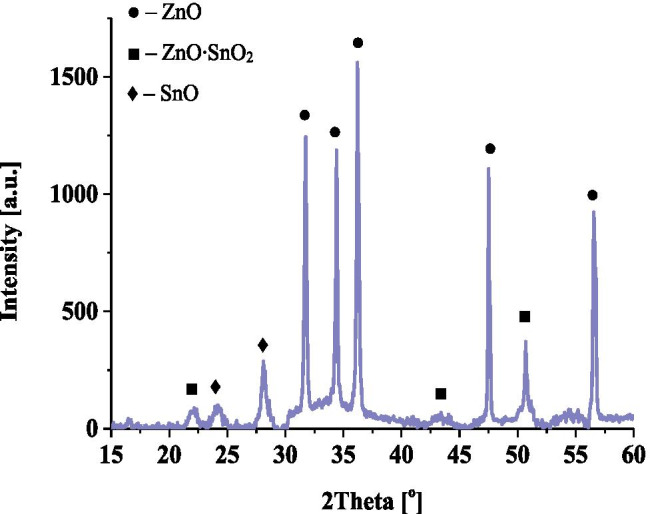
Diffractogram of ZnO-SnO_2_ NPs photocatalyst nanoparticles

Modification of the surface of ZnO NPs with tin(IV) oxide caused increase of its photocatalytic properties. On the basis of XRD analysis, the superstructure of zinc oxide on ZnO and the formation of a new 2ZnO∙SnO_2_ phase can be observed (Fig. 1). The forming crystalline phase provided a change in the energy gap of the material and thus caused efficient electron transfer between the conduction band and the valence band.

**Fig. 2:**
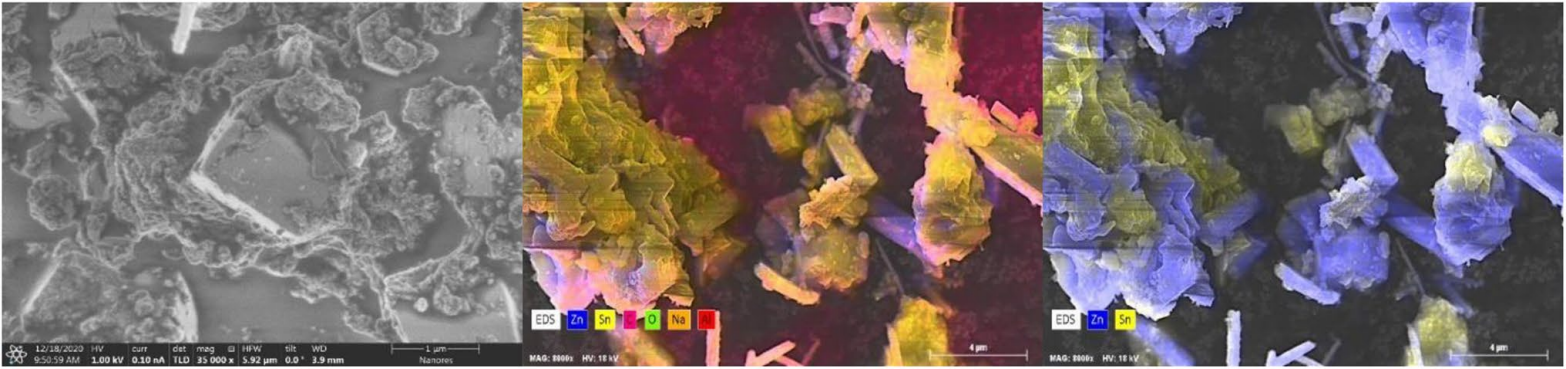
SEM–EDS micrographs of ZnO nanoparticles modified with tin(IV) oxide (ZnO-SnO_2_)

Figure 2 presents SEM microphotographs with EDS analysis of ZnO-SnO_2_ NPs. The analysis revealed the preparation of ZnO rods and a 2ZnO∙SnO_2_ phase characterised by a non-formed particle shape and a particle size of about 1–5 μm.

**Fig. 3:**
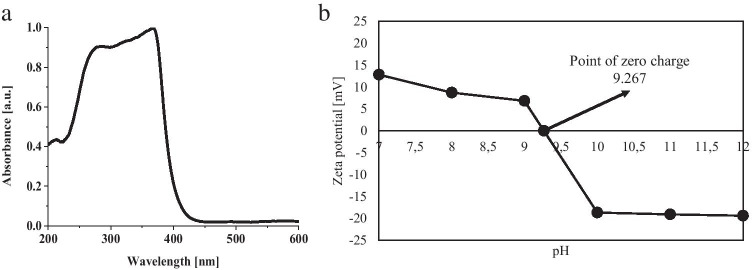
**a** Kubelka model for UV–Vis DRS analysis. **b** Zero-point charge fotokatalizatora ZnO-SnO_2_

The reflection spectrum of ZnO-SnO_2_ is shown in Fig. 3a. Based on the dependence of the Kubelka–Munk function, the width of the excited band was determined, which was 3.22 eV. The surface character of the photocatalyst was determined from the determined zero charge point (pzc) (Fig. 3b). If the pH of a suspended solid is above zero charge, it means that the surface is negatively charged and has the capacity to adsorb cations and participate in the cation exchange reaction (Fatehah et al. 2014), whereas when the pH is less than pzc, the surface is positively charged and has the ability to exchange anions. Processes carried out in neutral pH solutions result in the material having an affinity for neutral compounds and for anionic compounds. The photodegradation processes were carried out at neutral pH, hence a higher degree of degradation of the negative dyes: quinoline yellow and trypan blue were obtained.

#### Analysis of the photodegradation of dyes in the presence of ZnO-SnO_2_ NPs

To verify the photocatalytic activity of ZnO-SnO_2_, dyes differing in particle size, charge and structure were selected, i.e. methyl orange and trypan blue belonging to azo dyes, methylene blue belonging to azin dyes, rhodamine B as an example of a fluorescent dye and quinoline yellow a food dye. Under real conditions, a number of compounds are simultaneously present in the wastewater, making it necessary to take into account the interactions occurring between the compounds, which affect the overall removal efficiency of the system.

In the first step, the removal efficiencies of dyes from single-component systems were checked. Table 2 summarises the removal efficiencies of individual dyes after 60 min of photodegradation.

**Table 2:** Photodegradation efficiency of dyes in single-component systems

	Time [min]	0 (after 60 min of sorption)	15	30	45	60
MB	E_MB_ [%]	3.15	22.87	36.58	52.8	76.44
	R [mg/g]	0.31	2.28	3.65	5.27	7.63
RB	E_RB_ [%]	11.94	31.16	48.63	57.41	72.69
	R [mg/g]	1.55	4.04	6.31	7.45	9.43
TB	E_TB_ [%]	20.99	28.65	41.77	49.53	62.43
	R [mg/g]	9.97	13.61	19.84	23.53	29.66
MO	E_MO_ [%]	0.00	17.49	36.22	54.82	77.00
	R [mg/g]	0.00	1.56	3.24	4.90	6.88
YQ	E_YQ_ [%]	48.89	54.55	59.70	74.84	92.46
	R [mg/g]	5.30	5.92	6.48	8.12	10.03

**Fig. 4:**
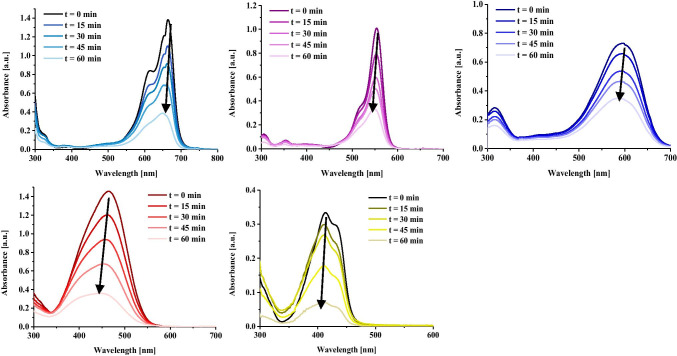
Photodegradation of selected dyes in the presence of ZnO-SnO_2_ NPs in single-component systems

**Fig. 5:**
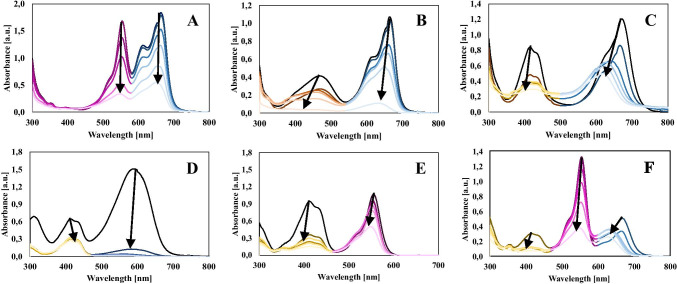
Photodegradation of selected dyes in the presence of ZnO-SnO_2_ NPs in binary and ternary systems after 60 min: A – mixture of RB with MB, B – mixture of MO with MB, C - mixture of YQ with MB, D - mixture of YQ with TB, E - mixture of YQ with RB, F - mixture of YQ, RB and MB

Among the dyes tested, the highest dye removal efficiency after photocatalysis was obtained for the degradation process of quinoline yellow, which value was 92.46% (Figs. 4 and 5). This is due to the fact that QY shows the lowest molecular weight, which resulted in increased adsorption of the dye on the catalyst surface (48.89%). Dye sorption is a key step in dye removal processes due to the surface nature of the photodegradation process. Other dyes were removed at a lower rate, but for all materials, the removal efficiency was above 60%. The reduced removal efficiency of TB is due to the increased initial concentration of the dye.

In the second stage, the efficiency of dye removal was verified in binary and ternary systems (Table 3).

**Table 3:** Photodegradation efficiency of dyes in multicomponent systems

	Time [min]	0 (after 60 min of sorption)	15	30	45	60
MB-RB	E_MB_ [%]	2.99	16.68	33.19	55.19	73.39
	E_RB_ [%]	0.36	18.36	39.59	69.11	80.91
	E_tot_ [%]	1.675	17.52	36.39	62.15	77.15
MB-MO	E_MB_ [%]	3.81	29.96	40.00	58.33	94.34
	E_MO_ [%]	35.83	39.29	44.73	62.52	92.42
	E_tot_ [%]	19.82	34.63	42.37	60.43	93.38
MB-YQ	E_MB_ [%]	25.61	52.77	65.06	74.06	81.67
	E_YQ_ [%]	43.77	55.63	54.76	58.73	66.56
	E_tot_ [%]	34.69	54.20	59.91	66.40	74.12
TB-YQ	E_TB_ [%]	91.93	97.90	99.03	99.43	99.76
	E_YQ_ [%]	52.64	55.06	52.15	53.28	60.39
	E_tot_ [%]	72.29	76.48	75.59	76.36	80.08
RB-YQ	E_RB_ [%]	12.57	10.07	18.90	27.67	55.29
	E_YQ_ [%]	63.74	76.6	70.16	79.89	85.50
	**E**_**tot**_** [%]**	38.16	43.34	44.53	53.78	70.40
YQ-RB-MB	E_YQ_ [%]	84.44	82.46	82.32	84.16	89.40
	E_RB_ [%]	2.67	11.73	26.12	46.15	72.80
	E_MB_ [%]	32.54	54.43	61.06	66.28	78.33
	E_tot_ [%]	39.88	49.54	56.50	65.53	80.18

In order to enable the photodegradation of compounds on the surface of the photocatalyst, it is necessary, at the first stage, to adsorb these compounds. The significance of sorption efficiency on the enhancement of photodegradation process was confirmed by analysis of variance (ANOVA) (Table 4).

**Table 4:** Analysis of variance of the effect of sorption efficiency on the photodegradation of dyes

	Sum square	df	Mean square	*F*	*p*
Regression	11,448.6	1	11,448.6	75.6186	0.0000
Rest	2422.4	16	151.40		
Total	13,871.0				

Based on the results obtained, 2 areas of different influence of sorption on the photodegradation of dyes can be observed. In systems where the sorption efficiency does not exceed, 5% of the degree of degradation of dyes is about 70%. In subsequent systems, increasing the sorption efficiency results in a gradual decrease in the photocatalysis effect, which is due to a decrease in the sample concentration in solution. However, if the sorption share is 20–30%, the influence of the photodegradation effect increases again. Two different mechanisms can be observed here. On the one hand, an increase of the sorption effect results in a decrease of the total dye concentration that can be removed by photodegradation. The opposite effect is a favourable correlation due to the surface nature of the photodegradation process. An increase in the number of sorbed pigment particles increases the efficiency of the photodegradation process, which confirms the following phenomena (Lee et al. 2019; Rodwihok et al. 2021). Based on the results obtained, it can be concluded that it is advantageous for the photocatalytic material to be characterised by sorption properties, but the process should be physical in nature, so that photodegradation can take place and decomposition residues can be removed from the material surface.

**Fig. 6:**
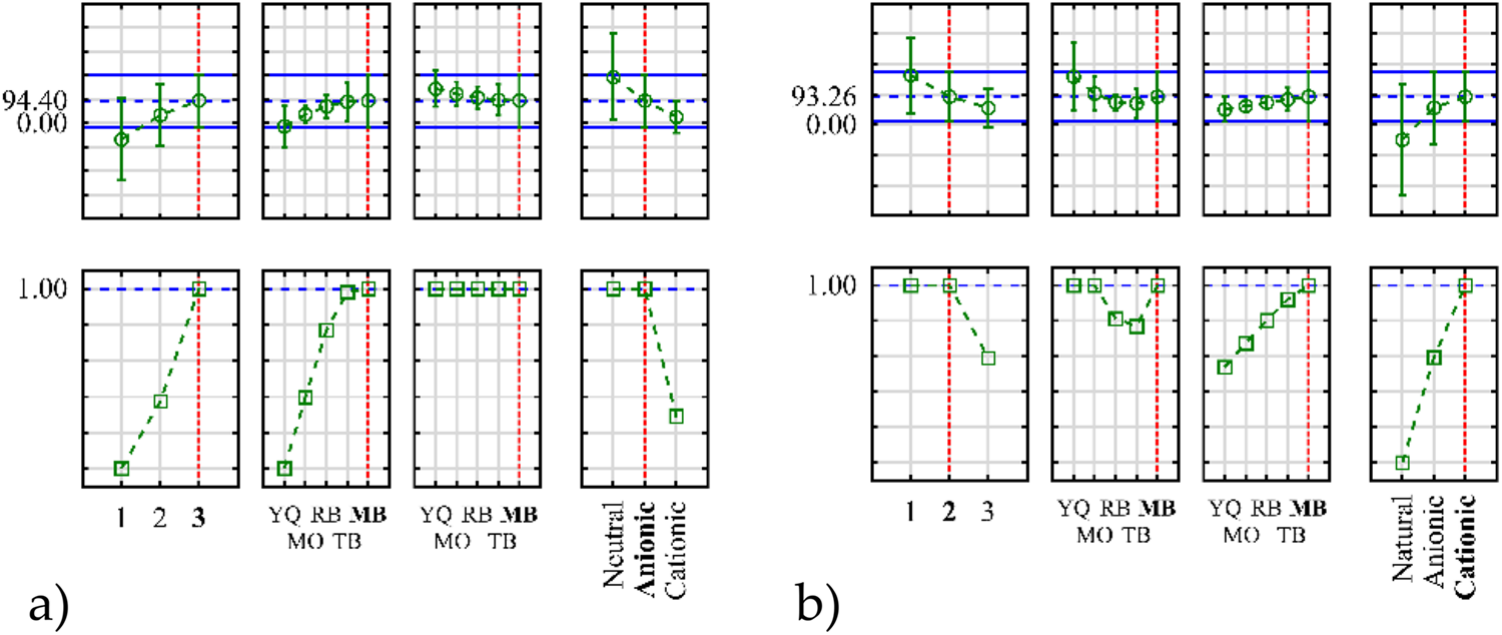
**a** Influence of dye properties on their sorption efficiency and **b** influence of dye properties on their photodegradation efficiency

Analysis of the influence of parameters on the sorption efficiency showed a significant effect of the amount of components in the system, the influence of the type and nature of the dye to be removed (Fig. 6). While keeping the concentration of dyes in the system constant, increasing the amount of components favourably affects their sorption on the material surface. Dyes of different structures gradually fill the surface of the catalyst, which makes it possible to obtain a higher degree of their uptake from the solution. The presence of other dyes in the system does not significantly impair the sorption properties. Based on the determined isoelectric point, the cationic character of the catalyst surface was confirmed. Due to the positively charged surface, higher sorption efficiency was observed for the anionic and neutral compounds, while significantly lower sorption efficiency was observed for the cationic dyes (Jose et al. 2021). In the case of the photodegradation process, an opposite relationship was observed. The cationic dyes are more easily separated from the catalyst surface, and the removal of the degradation residues from the positively charged surface is facilitated.

### Analysis of the kinetics of dye degradation in the presence of a photocatalyst

The decomposition rates of dyes in aqueous solutions in the presence of a photocatalyst were compared with the kinetics of photodegradation processes proposed by Langmuir–Hinshelwood (L–H):

3 $$lnC=\mathrm{ln}{C}_{0}-kt$$

where $k$ is the photodegradation rate constant $(mi{n}^{-1}$) and ${C}_{0}$ is the initial dye concentration.

For the material with the highest photodegradation efficiency, i.e. ZnO-SnO_2_, the kinetics of decomposition of dyes, i.e. one-, two- and three-component mixtures, was studied. For this purpose, 2 ml of the suspension was taken from the system every 15 min, swabbed and analysed by spectrophotometry in the range from 300 to 1000 nm. The results are presented in Table 5. The decomposition rate constants of the dyes for single-component systems ranged from 0.0122 to 0.0295 min^−1^. In multicomponent systems, a decrease in the decomposition rate constant for quinoline yellow was observed. This is due to the blocking of the photocatalyst surface by the other components with higher molecular weight, limiting the sorption of YQ.

**Table 5:** Langmuir–Hinshelwood kinetic parameters for photodegradation of selected dyes onto ZnO-SnO_2_

		k [min^−1^]	C_0_ [mg/dm^3^]	C_0,ex_ [mg/dm^3^]	*R*^2^
MB	MB	0.0221	21.50	19.33	0.9203
YQ	YQ	0.0295	14.79	11.09	0.7930
MO	MO	0.0236	20.29	17.87	0.9351
RB	RB	0.0188	23.50	22.86	0.9824
TB	TB	0.0122	78.67	75.07	0.9715
RB-MB	RB	0.0285 0.0214	8.85	7.62	0.9578 0.9443
	MB		9.40	8.31	
MO-MB	MO	0.0114 0.0178	6.88	6.41	0.7019 0.7542
	MB		9.56	9.62	
YQ-MB	YQ	0.0074 0.0227	5.26	5.45	0.8846 0.9934
	MB		3.80	4.00	
YQ-TB	YQ	0.0021 0.0556	3.59	3.53	0.4238 0.9739
	TB		1.95	2.53	
YQ-RB	YQ	0.0132 0.0104	3.89	4.01	0.7839 0.7422
	RB		4.80	4.22	
YQ-RB-MB	YQ	0.0081 0.0203 0.0171	0.66	0.60	0.4304 0.8761 0.9579
	RB		6.84	5.82	
	MB		1.50	1.56	

## Conclusion

In the paper, the decomposition analysis of dyes from mono-, bi- and tri-component systems using ZnO-SnO_2_ NPs as photocatalyst has been reported. After 60 min of processes, dye removal efficiencies of 76.44, 72.69, 62.43, 77.00 and 92.46% were obtained for the degradation of methylene blue, rhodamine B, trypan blue, methyl orange and quinoline yellow, respectively. For binary and ternary systems, dye removal efficiencies for all processes exceeded 70%. The presence of components of the same nature did not cause a decrease in the decomposition rate of the dyes. Significant differences were observed for compounds differing in particle size, which may be due to blocking of surface access and competition of the components for the catalyst active sites. The removal efficiency of cationic dyes in the presence of neutral dyes and anionic dyes significantly decreases due to charge imbalance on the material surface.

## Data Availability

All the data and tools/models used for this work are publicly available.
